# Haemodynamic determinants of quality of life in chronic heart failure

**DOI:** 10.1186/s12872-022-02829-w

**Published:** 2022-09-16

**Authors:** Serlie Fatrin, Nduka C. Okwose, Kristian Bailey, Lazar Velicki, Dejana Popovic, Arsen Ristic, Petar M. Seferovic, Guy A. MacGowan, Djordje G. Jakovljevic

**Affiliations:** 1 Cardiovascular Research Theme, Translational and Clinical, and Biosciences Research Institutes, Medical School, Faculty of Medical Sciences, Newcastle University, William Leech Building 4th Floor, Framlington Place, Newcastle upon Tyne, NE2 4HH UK; 2 Newcastle upon Tyne Hospitals NHS Foundation Trust, Newcastle upon Tyne, UK; 3 Research Centre (CSELS), Institute for Health and Wellbeing, Faculty of Health and Life Scurnces, Coventry University, Coventry, UK; 4 Institute of Cardiovascular Diseases Vojvodina, University of Novi Sad, Novi Sad, Serbia; 5 Department of Cardiology, Medical School, Clinical Centre of Serbia, University of Belgrade, Belgrade, Serbia

**Keywords:** Heart failure, Quality of life, Exercise haemodynamics

## Abstract

**Background:**

Heart failure patients demonstrate reduced functional capacity, hemodynamic function, and quality of life (QOL) which are associated with high mortality and morbidity rate. The aim of the present study was to assess the relationship between functional capacity, hemodynamic response to exercise and QOL in chronic heart failure.

**Methods:**

A single-centre prospective study recruited 42 chronic heart failure patients (11 females, mean age 60 ± 10 years) with reduced left ventricular ejection fraction (LVEF = 23 ± 7%). All participants completed a maximal graded cardiopulmonary exercise test with non-invasive hemodynamic (bioreactance) monitoring. QOL was assessed using Minnesota Living with Heart Failure Questionnaire.

**Results:**

The average value of QOL score was 40 ± 23. There was a significant negative relationship between the QOL and peak O_2_ consumption (r = − 0.50, *p* ≤ 0.01). No significant relationship between the QOL and selected exercise hemodynamic measures was found, including peak exercise cardiac power output (r = 0.15, *p* = 0.34), cardiac output (r = 0.22, *p* = 0.15), and mean arterial blood pressure (r = − 0.08, *p* = 0.60).

**Conclusion:**

Peak O_2_ consumption, but not hemodynamic response to exercise, is a significant determinant of QOL in chronic heart failure patients.

## Introduction

Exercise intolerance is a clinical hallmark of chronic heart failure. Reduced cardiac output and oxygen extraction are main underlying pathophysiological mechanisms explaining exercise intolerance in chronic heart failure [1–3]. Functional capacity (represented by peak exercise oxygen consumption) and hemodynamic response to exercise (peak exercise cardiac output and cardiac power output) are strong determinants of prognosis and can be used to improve risk stratification in chronic heart failure [4, 5]. Reduced functional capacity and cardiac function are major causes of morbidity and mortality in chronic heart failure [6].

Patients with chronic heart failure demonstrate significantly lower Quality of Life (QOL) in comparison with other chronic conditions [7]. Improvement of QOL is an important end point in clinical and research practice [8, 9]. Despite this, limited number of studies have attempted to identify clinical and physiological determinants of QOL in chronic heart failure. In particular, association between hemodynamic function during exercise represented by peak cardiac power output, a marker of overall function and pumping capability of the heart [4], has not been evaluated. Therefore, the aim of the present study was to assess functional capacity and hemodynamic response to exercise in chronic heart failure and determine their relationship with QOL.

## Methods

A prospective, single-centre, cross-sectional study was performed to assess functional capacity, hemodynamic response to exercise in chronic heart failure patients. Quality of life was also assessed as detailed below. A total of 42 patients with stable chronic heart failure due to reduced left ventricular ejection fraction were recruited into the study. Patients were additionally screened to ensure that they met study inclusion criteria, which included heart failure due to reduced left ventricular ejection fraction (LVEF < 40%), NYHA functional class II and III, age 50 years or more, clinically stable (on optimal medication, no hospitalisation, or any acute event of worsening heart failure) for at least 6 weeks prior to the study, and an ability to perform maximal graded cardiopulmonary exercise stress testing.

All participants were instructed not to eat two hours before the visit to the clinical research facility where assessment procedures were undertaken. Participants were also instructed not drink alcohol or caffeine on the day of assessment. Eligible participants signed an informed, written consent and all study procedures were in accordance with the Declaration of Helsinki and meets international ethical standards [10]. The study was approved by the research ethics committee of the National Health Service Northeast England—Tyne and Wear South, and local Research and Development department.

Upon arrival at the Clinical Research Facility, study participants’ anthropometric measurements (body weight and height) were obtained. Participants also answered a standardized health screening questionnaire according to the Standard Operating Procedure of the Clinical Exercise Laboratory. All patients completed a maximal graded cardiopulmonary exercise test using a semi-recumbent, electromagnetically controlled cycle ergometer (Corival, Lode & Groningen, Netherland) with non-invasive gas exchange (Cortex metalyser 3B, Leipzig, Germany) and bioreactance method for haemodynamic monitoring (NICOM®, Cheetah Medical, USA) [11]. Exercise stress test was carried out according to recommendations from the American College of Sports Medicine and was supervised by a consultant cardiologist with special interest in heart failure who was part of the research team. Before exercise testing, implanted devices were turned off for patients with ICD. Test protocol included a warm-up for 3 min at 20 W and then load was increased by 10 W per minute until maximum exertion was achieved. The cadence of 60–70 revolutions per minute was maintained throughout the test. Hemodynamic (cardiac output and stroke volume), arterial blood pressure, electrocardiogram and gas exchange measurements were performed at rest and during exercise test. Standardised Borg scale (0–20) was used to indicate level of exertion [12] Maximal effort was achieved if: (1) the participant could no longer cycle at 60–70 revolutions per minute, (2) respiratory exchange ratio of 1.10 or higher, (3) oxygen consumption does no further increase despite increase in workload, or (4) ≥ 90% of maximum age adjusted heart rate. The test was terminated at maximum exertion or if the participant had requested to terminate the exercise test earlier.

Hemodynamic variables measured during exercise stress test were heart rate, systolic and diastolic blood pressure, stroke volume, which are then used to calculate cardiac output, mean arterial blood pressure, and cardiac power output. Cardiac power output was calculated using the following formula:

$${\text{CPO}} = \left( {{\text{CO}} \times {\text{MAP}}} \right) \times {\text{K}}$$

where CPO is cardiac cpower output, CO is cardiac output, MAP is mean arterial blood pressure and K = 2.22 × 10^–3^, is the conversion factor into watts [13].

Non-invasive gas exchange measurement allowed determination of peak oxygen consumption and subsequent calculation of the arteriovenous oxygen difference using measured oxygen consumption and cardiac output. Minnesota Living with Heart Failure (MLHF) Questionnaire was used to assess heart failure related QOL.

All results are expressed as mean ± SD unless stated otherwise. Before SPSS analysis, the data were screened for univariate and multivariate outliers using Z-distribution cut-offs and Mahalabonis distance test. Kolmogorov Smirnov test was used to assess normality. Baseline haemodynamic measures and response to exercise were compared between patients and healthy controls using independent sample t-test. Pearson’s coefficient of correlation (r) was used to assess the relationship between quality of life and measures of hemodynamic response to stress exercise in chronic heart failure. A *p* value < 0.05 was considered statistically significant. Statistical analysis was carried out using SPSS version 24.0 (SPSS Inc, Chicago, IL).

## Results

Demographic, clinical, and physical characteristics of patients are indicated in Table 1.

**Table 1:** Participants’ demographic and clinical characteristics (n = 42)

Age (years)	60 ± 10
Male/female (%)	74/26
Weight (kg)	88 ± 15
Height (m)	1.73 ± 0.07
BMI (kg/m^2^)	29 ± 4
Functional class (NYHA) II/III	19/23
LVEF (%)	23 ± 7
MLHF	40 ± 23
Aetiology (IHD/DCM/Others)	22/14/6
AF	14
ICD	19
*Medication; n (%)*	
ACE inhibitors	27 (64)
β-blockers	41 (98)
ARBs	9 (21)
Diuretics	33 (78)
MRA	27 (64)
Anti-arrhythmic	14 (33)
NSAID/analgesic	12 (28)
Antiplatelet/anticoagulant	17 (40)
ARNI	4 (9)

*ACE* angiotensin converting enzyme, *AF* atrial fibrillation, *ARB* angiotensin receptor blockers, *ARNI* angiotensin receptor neprilysin inhibitor, *DCM* dilated cardiomyopathy, *IHD* ischaemic heart disease, *LVEF* left ventricular ejection fraction, *MLHF* Minnesota living with heart failure, *MRA* mineralocorticoid receptor antagonist, *NYHA* New York Heart Association, *NSAID* non-steroid anti-inflammatory drugs

**Fig. 1:**
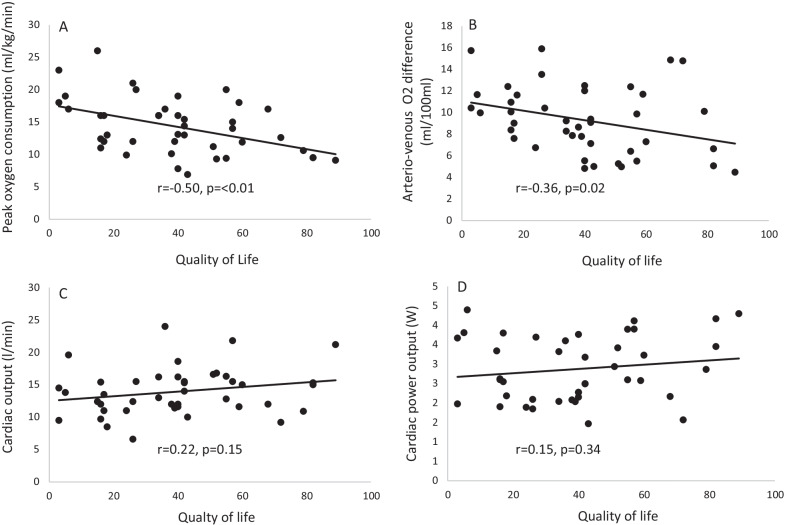
Relationship between quality-of-life score and metabolic and haemodynamic response to exercise in chronic heart failure patients: **A** peak oxygen consumption, **B** peak arteriovenous oxygen difference, **C** peak cardiac output, **D** peak cardiac power output

There was a higher proportion of males (n = 31, 74%) and patients were categorised into NYHA functional class II (n = 19) or III (n = 23). Metabolic and haemodynamic measures at rest and peak exercise are presented in Table 2. Pearson’s coefficient of correlation was used to assess the strength of the relationship between hemodynamic and metabolic response to exercise and QOL. There was a significant negative relationship between QOL score and peak oxygen consumption (r = − 0.50, *p* < 0.01, Fig. 1A). Peak exercise arteriovenous oxygen difference was also significantly related to QOL (r = − 0.36, *p* = 0.02, Fig. 1B). There was no relationship between QOL score and peak exercise cardiac output, cardiac power output Fig. 1C–D), or mean arterial blood pressure (*p* > 0.05).

**Table 2:** Metabolic and haemodynamic measures at rest and peak exercise (n = 42)

Parameter	Rest	Peak exercise
Oxygen consumption (ml/kg/min)	4.1 ± 1.2	14.1 ± 4.7
Systolic blood pressure (mmHg)	109 ± 19	133.6 ± 32.6
Diastolic blood pressure (mmHg)	69 ± 10	74.4 ± 13.2
Mean arterial blood pressure (mmHg)	83 ± 12	88 ± 22
Stroke volume (ml/beat)	87 ± 23	127 ± 35
Cardiac output (l/min)	6.0 ± 1.3	13.9 ± 3.6
Cardiac power output (watts)	1.1 ± 0.3	2.9 ± 0.8
Heart rate (beats/min)	70 ± 9	106 ± 21
Arteriovenous oxygen difference (ml/100 ml)	6.4 ± 2.7	9.2 ± 3.3

## Discussion

The major findings of the present study suggest that in chronic heart failure, QOL is significantly correlated with peak oxygen consumption mediated primarily via improved arterio-venous oxygen difference (increased skeletal muscle oxygen uptake) but not exercise hemodynamic measures such as cardiac power output, cardiac output, or mean arterial blood pressure. These findings provide better understanding of determinants of QOL in chronic heart failure, further suggesting that impaired cardiac function may not consequently lead to reduced quality of life. On the other hand, patients with higher functional capacity (peak O2 consumption) will likely have better QOL as demonstrated with lower scores on Minnesota Living with Heart Failure quality of life questionnaire.

Cardiac power output has been proposed to be the best indicator of overall function and pumping capability as it accounts for both flow and pressure generating capacities of the heart [5, 14]. When compared to result of studies with age matched controls [5, 14], findings of the present study reaffirms that patients with chronic heart failure showed reduced cardiac function as represented by lower resting and peak exercise cardiac power output.

Reduced exercise capacity (i.e., lower peak exercise O_2_ consumption) reported in the present study is consistent with previous studies [15–18] with reduced cardiac output and stroke volume being the main cardiac determinants responsible for lower exercise tolerance [19]. Although, being a strong prognostic marker in chronic heart failure, oxygen consumption is not only influenced by central (cardiac) but also by peripheral factors (skeletal muscle function), systemic inflammation, ageing, motivation to exercise, gender [1, 2]. Like the present study, previous reports have shown a lack of relationship between central haemodynamic measures and exercise tolerance [9, 20]. In contrast, exercise tolerance assessed using a series of self-paced corridor walk tests showed moderate correlations with cardiac index [20], thus- questioning the assessment of cardiac output in heart failure patients using maximal tests performed in the laboratory which do not represent patients’ true capabilities.

Despite reduced peak oxygen consumption compared to healthy subjects, our results demonstrate a significant negative relationship between QOL score and peak oxygen consumption like other reports [21, 22]. Peripheral blood flow blood is a better determinant of exercise capacity. An increase in muscle oxygen extraction beyond submaximal exercise has a compensatory effect on reduced cardiac output, to increase peak oxygen consumption [23–25]. Hence, we can infer that an increase in peak arterio-venous oxygen difference, but not cardiac output or cardiac power output is the reason for significant correlation between QOL and peak oxygen consumption.

Numerous instruments (questionnaires) have become available to measure patients’ health-related quality of life. However, disease-specific questionnaires such as Minnesota Living with Heart Failure is more sensitive to detecting changes than generic questionnaires [26]. In the present study the average score of MLHF quality of life questionnaire was 40, with range from 3 to 89, suggesting wide range of quality of life in the studied patients.

Quality of life is increasingly becoming one of the primary outcomes in clinical and research practice in heart failure. Pharmacological, surgical, and physiological interventions known to improve functional capacity are likely to lead to improved QOL in chronic heart failure, while those focusing in improving haemodynamic function only may not necessarily lead to patient experience of better QOL.

The following limitations of the current study should be taken into consideration. Firstly, the number of patients recruited into the study was only moderate. Secondly, majority of patients were males with less than one third of patients were females. Lastly, this was a cross-sectional study which evaluated exercise capacity using only one method. Therefore, generalisability of the present study findings and its conclusions should be considered with caution.

In conclusion, patients with chronic heart failure demonstrate reduced functional capacity and overall cardiac function. Significant negative relationship between functional capacity and quality of life score suggests that peak exercise oxygen consumption mediated via increased skeletal muscle oxygen consumption is an important determinant of quality of life in chronic heart failure. In contrast, peak exercise central hemodynamic measures i.e., cardiac power output, cardiac output and mean arterial blood pressure were not significantly correlated with quality-of-life score, indicating their limited capacity to reflect quality of life in patients with chronic heart failure.

## Data Availability

All data generated or analysed during this study are included in this published article. Additional information will be made available upon reasonable request by the corresponding author.
